# Supplementary material for: Minmers are a generalization of minimizers that enable unbiased local Jaccard estimation
Source: Bioinformatics. 2023 Aug 21;39(9):btad512. doi: 10.1093/bioinformatics/btad512 (PMC10505501; doi:10.1093/bioinformatics/btad512)
Supplement: btad512_Supplementary_Data [file btad512_supplementary_data.pdf]

Minmers are a generalization of minimizers that enable unbiased local Jaccard estimation

July 19, 2023

1 Supplementary Materials

1.1 Probabilistic filtering for the minhash

We construct a predictor, Y_i, of the numerator of the minhash formula for A and B_i conditioned on the size of the intersection $|\pi_s(A) \cap \pi_s(B_i)|$. This predictor generates a probability distribution for the ANI of a candidate mapping without needing compute the expensive $\pi_s(A \cup B_i)$ step. We start by dividing $\pi_s(A) \cup \pi_s(B_i)$ into two parts where $C_i = \pi_s(A) \cap \pi_s(B_i)$ and $G_i = (\pi_s(A) \cup \pi_s(B_i)) \setminus C_i$ resulting in two sets of size c_i and $2s - c_i$, respectively. The problem can now be formulated as follows: what is the probability that y elements from C_i are also part of the sketch $\pi_s(A \cup B_i)$?

Leveraging the fact that $\pi_s(A \cup B_i) = \pi_s(\pi_s(A) \cup \pi_s(B_i))$ and that all orderings of elements in $\pi_s(A \cup B_i)$ are equally likely, we can view the problem as assigning the c_i shared elements to $2s - c_i$ slots, where the first s slots are considered as a "success" and the remaining $s - c_i$ slots are considered as a "failure" (Supplementary Figure 1).

We have the following formulas:

$$
\begin{aligned}
\Pr(Y_i = y | c_i) &= \text{Hypergeom}_{pdf}(2s - c_i, s, c_i, y) \\
&= \frac{\binom{s}{y}\binom{s-c_i}{c_i-y}}{\binom{2s-c_i}{c_i}} \\
\Pr(Y_i \leq y | c_i) &= \text{Hypergeom}_{cdf}(2s - c_i, s, c_i, y) \\
&= \sum_{i=0}^{y-1} Pr(Y_i = y | c_i)
\end{aligned}
$$

Let $z = \arg\max_i c_i$ be a position with the maximum intersection size over all B_i, i.e. the position in B that overlaps with the most selected minmer intervals. We can now find a minimum intersection size τ such that for any $c_i < \tau$,

$$\Pr(\hat{J}(A, B_i) > \hat{J}(A, B_z) - \Delta_J) < 1 - \delta$$

where Δ_J is the difference in the Jaccard that corresponds to an ANI value Δ_{ANI} less than the ANI value predicted by $\hat{J}(A, B_z)$ and δ is a desired confidence level. To calculate this probability, we can use the following summation

$$\begin{aligned}\Pr(\hat{J}(A, B_i) > & \hat{J}(A, B_z) - \Delta_J) \\ & = \sum_{y=0}^{s} \Pr(Y_i = y \mid c_i) \Pr(Y_z < y + \Delta_J \mid c_z)\end{aligned}$$

For each intersection size, we can identify a cutoff in $\mathcal{O}(s \log(s))$ time. As a preprocessing step, we compute cutoffs for each of the s possible intersection sizes at the indexing stage. Candidate regions that are unlikely to have an ANI within Δ_{ANI} of the best predicted ANI are then pruned. The default Δ_{ANI} and δ confidence parameters of MashMap3 are 0 and 0.999, respectively, as in many cases the lower scoring mappings for a segment are filtered out by the plane-sweep filtering method of MashMap described in Jain *et al.* (2018).

We compute two passes over the interval endpoints in L. In the first pass of stage 1, the maximum intersection size c_z is obtained. In the second pass, candidate mappings whose intersection is above the cutoff derived from c_z are obtained. Consecutive candidate mappings are grouped into candidate regions and passed to stage 2.

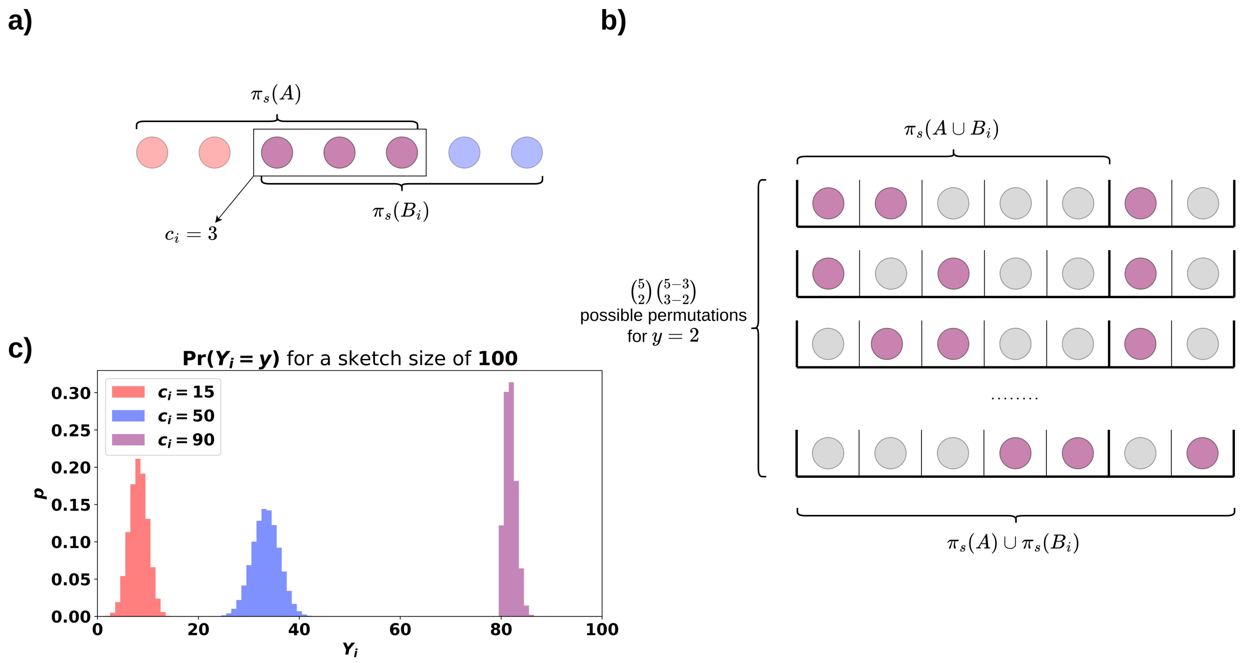

Supplementary Figure 1: **Estimating the minhash from $\pi_s(A)$ and $\pi_s(B_i)$.** **(a)** Given two sketched sets $\pi_s(A)$ and $\pi_s(B_i)$, we can compute the size of their intersection c_i. **(b)** By considering $C_i = \pi_s(A) \cap \pi_s(B_i)$ as purple balls and $G_i = (\pi_s(A) \cap \pi_s(B_i)) \setminus C_i$ as grey balls, we can enumerate all possible permutations of their union such that exactly y purple balls fall within the first s slots. **c)** The distribution of the minhash numerator Y_i for different values of c_i when $s = 100$. The corresponding distribution of the minhash can be obtained by dividing Y_i by the sketch size s.

1.2 Efficiently computing the rolling minhash

To keep track of the rolling minhash for a candidate region, MashMap3 uses an array $V = (-1, 0, 0)$, (x_1, α_1, β_1), (x_2, α_2, β_2), ..., (x_s, α_s, β_s) where each x_j represents one of the s minmer hash values from $\pi_s(A)$ in increasing order and for each $i \in [a, z)$, the values α_j and β_j are

- $\alpha_j = 1 \quad \text{if} \quad x_j \in \pi_s(B_i) \quad \text{else} \quad 0$
- $\beta_j = 1 + |\{x \in \pi_s(B_i) \text{ s.t. } x_{j-1} < x < x_j\}|$

We can imagine V as a set of s buckets labeled by the s corresponding hash values of A and sorted in increasing order. At each position $i \in [a, z)$, each bucket j holds x_j and all $\beta_j - 1$ reference minmers in $\pi_s(B_i)$ between x_j and x_{j-1}. A bucket is marked "good" ($\alpha_j \to 1$) if $x_j \in \pi_s(B_i)$. It remains to find the largest integer p_i such that the number of minmers in the first p_i buckets is at most s. Given p_i, the numerator of the minhash formula, Y_i, is the number of "good" buckets in the first p_i buckets.

For a candidate region $[a, z)$, we initialize V by inserting all of the minmers from the reference index whose intervals overlap with a and set

$$p_a = \max_q \left(\sum_{j=0}^{j \le q} \beta_j \le s \right)$$

It follows that $Y_a = \sum_{j=1}^{j \le p_a} \alpha_j$

In order to keep track of intervals which overlap with the current position, we use a min-heap H sorted on interval endpoints. We then continue to iterate through minmer intervals from the reference in order based on their start points, stopping once the intervals no longer overlap with $[a, z)$. For each minmer interval starting at $i \in [a + 1, z)$, we pop intervals from H that end at or before i. For each interval popped from H, we update V in $\mathcal{O}(\log(s))$ time through a binary search, decrementing the corresponding β_j and setting $\alpha_j = 0$ if the interval represents a shared minmer. The new interval is added in a similar manner and the necessary α and β values are updated. After V is updated, p_i is updated from p_{i-1} by incrementing or decrementing until it is the maximal value such that $p_i = \max_q \left(\sum_{j=0}^{j \le q} \beta_j \le s \right)$. By keeping track of p_{i-1} and the sums $\sum_{j=0}^{j \le p_{i-1}} \beta_j$ and $\sum_{j=0}^{j \le p_{i-1}} \alpha_j$, the new p_i and corresponding sums are updated in constant time per window.

While the MashMap3 implementation of the second filtering stage still requires $\mathcal{O}(\log(s))$ time to update the minhash for each sliding window within the candidate region, it is significantly more efficient than MashMap2's ordered map in practice due to V being a static data structure in contiguous memory, only requiring updates to counters.

1.3 Minmer density

To obtain the density of the minmer scheme, we inspect how the rank of a k-mer changes with each sliding window. In particular, we use the rank of the k-mer in its first and last windows, i.e. the windows in which the k-mer is just entering and just about to leave. To inspect this, we characterize the distribution of the first rank, the distribution of the final rank given the first rank, and the probability of the rank ever being

less than or equal to s given the first and last ranks.

Let S be a sequence of $2w-1$ uniformly random numbers in $[0,1]$. We denote the middle element at position w as z, its rank in the leftmost window of size w as r_1, and its rank in the rightmost window of size w as r_w. Let C_{r_1,r_w} be a conditional indicator r.v. such that $\Pr(C=1|r_1,r_w)=\Pr(C_{r_1,r_w}=1)$ where $C=1$ only if there exists a window of length w in S such that the rank of z in the window is at most s. This event corresponds to the element z being a minmer.

Lemma 1.1.

$$\Pr(C_{r_1,r_w}) \quad = \begin{cases} \sum_{u=0}^{\delta} \Pr(U=u)\frac{\binom{2u+r_w-r_1}{u+r_w-s}}{\binom{2u+r_w-r_1}{u}} & r_1>s, r_w>s \\ 1 & otw \end{cases}$$

where $U \sim \text{Hypergeometric}(w-1, r_1-1, w-r_w)$ and $\delta=\min(r_1-1, w-r_w)$.

Proof. Given the initial rank r_1 and the final rank r_w, we can model the path of the rank as left and right unit steps on a number line starting at point r_1 and ending at r_w. At each step in this path, the rank either increases, decreases, or remains the same. The event C_{r_1,r_w} is then equivalent to the event that the path touches the point s on the axis. Let $\omega=\omega_{\text{left}}z\omega_{\text{right}}$ be a sequence of length $2w-1$ representing the elements in S. We let $\omega_{left}=ppqpq...$ and $\omega_{\text{right}}=qpqppq...$ where each element is labeled as p if it is less than z and q otherwise. We define x and y as the number of ps and qs in ω_{left}, respectively, and similarly a and b are the number of ps and qs in ω_{right}, respectively. At step i, the rank z can decrease only if $\omega_{\text{left}}[i]=p$ and $\omega_{\text{right}}[i]=q$. Similarly, the rank will increase only if $\omega_{\text{left}}[i]=q$ and $\omega_{\text{right}}[i]=p$. Otherwise, the rank will remain the same. We note that there can be no more than $\max(r_1-1, w-r_w)$ left steps, as $x=r_1-1$ and $b=w-r_w$.

For each of the x ps in ω_{left}, we sample without replacement from ω_{right}. By considering each sampling of a q as a success, we see that the number of left steps given the initial and final ranks r_1 and r_w can be modeled as a hypergeometric random variable $U \sim \text{Hypergeometric}(w-1, x, b)$.

With a set of u left steps, we can calculate the number of right steps v by observing that if we have u pq pairs, then there must be $x-u$ pp pairs, $b-u$ qq pairs, and therefore $y-(b-u)=r_w-r_1+u$ qp pairs. Given a set of u left steps and v right steps, there are $\binom{u+v}{u}$ total paths. Of these paths, we aim to find the ones which touch point s on the axis. Using the reflection principle Comtet (1974), we observe that there are $\binom{u+v}{u+r_w-s}$ such paths and therefore

$$\frac{\binom{u+v}{u+r_w-s}}{\binom{u+v}{u}} = \frac{\binom{2u+r_w-r_1}{u+r_w-s}}{\binom{2u+r_w-r_1}{u}}$$

□

With the conditional distribution C_{r_1,r_w} at hand, we can define the marginal distribution of C.

Theorem 1.2.

$$\Pr(C=1) = \frac{1}{w} \sum_{r_1, r_w \in \{1 \dots w\}^2} \Pr(C=1|r_1, r_w) \Pr(R_w = r_w | r_1)$$

Where $R_1 \sim \text{Uniform}\{1, w\}$ and $R_w|r_1 \sim \text{BetaBinomial}(r_1, w - r_1 + 1)$ are random variables for the first and last rank of z, respectively.

Proof. Given r_1, the initial rank of z, we can use order statistics for uniform distributions to infer that the value of z is sampled from a Beta distributed r.v. $Z \sim \text{Beta}(r_1, w - r_1 + 1)$. Given the value z, we can predict the final rank of z by considering the remaining $w-1$ elements as Bernoulli trials each with probability z of having a lower value than z. Therefore, we have that $R_w|z \sim \text{Bin}(w-1, z)$. We can obtain the marginal of R_w via

$$\Pr(R_w = r_w) = \int_0^1 \Pr(R_w = r_w|p) \Pr(Z = z) dp$$

which is the Beta-binomial distribution with $n = w - 1$, $\alpha = r_1$ and $\beta = w - r_1 + 1$. □

1.4 Minmer interval density

We will prove the density of minmer intervals in a similar fashion to the proof for minimizers. We define a window of length w as at position i as W_i and say W_i is *charged* if $\pi_s(W_i) \neq \pi_s(W_{i-1})$. Like minimizers, the set of minmers between two adjacent windows can differ by at most one, as only a single minmer can leave the sketch at a time. Unlike minimizers, though, it is possible for a k-mer at position i to charge multiple windows by exiting and then re-entering the sketch. Therefore, the number of charged windows in a sequence is at least the number of minmers.

Consider a super-window of $w+1$ k-mers starting at position $i-1$ and let $\pi_s(W_i \cup W_{i-1})$ be the lowest s k-mers in the super-window. W_i is then not charged if and only if both $x_{i-1} \notin \pi_s(W_i \cup W_{i-1})$ and $x_{i+w-1} \notin \pi_s(W_i \cup W_{i-1})$. Assuming each position is equally likely to be part of the sketch, the probability of the first and last k-mers not being in the sketch is $\binom{w-1}{s}/\binom{w+1}{s}$ and therefore the probability that W_i is charged is

$$\begin{aligned} \Pr(W_i \text{ is charged}) &= 1 - \frac{\binom{w-1}{s}}{\binom{w+1}{s}} \\ &= 1 - \frac{(w-s+1)(w-s)}{w(w+1)} \end{aligned}$$

Assuming independence over windows, we have that the density of charged windows is equal to the probability that any window is charged and therefore the density of minmer intervals is $1 - \frac{(w-s+1)(w-s)}{w(w+1)}$.

1.5 Minmer spread

We now turn our attention to characterizing the distribution of distances between adjacent minmers using a proof described in joriki (2012).

Consider a window of length $w + 1$ which contains s sampled k-mers and is anchored at the left-most sampled k-mer. Assuming a set of $w+1$ unique k-mers , we have that each of the $w+1$ k-mers is equally likely to be sampled. Let $X_1, ..., X_{s-1}$ be a set of integers randomly sampled from $\{1, ..., w\}$ such that $X_i < X_{i+1}$. We define the distance between X_i and X_{i+1} as $G_i = X_{i+1} - X_i$. We let $X_0 = 0$ represent the first k-mer in the window positioned at the first location.

Lemma 1.3. $\Pr(G_i = d) = \frac{\binom{w-d}{s-2}}{\binom{w}{s-1}}$

Proof. Let us consider our $w + 1$ unique sorted integers arranged on a circle instead of a line. We then "cut" the circle at any one of the s sampled integers and renumber the w remaining integers starting from 1 after the cut. There are now $s-1$ integers uniformly sampled from $\{1, ..., w\}$. By fixing the first sample at position d and enforcing that all $s - 2$ remaining integers are sampled from $\{d + 1, ..., w\}$, we see that there are $\binom{w-d}{s-2}$ such samples. Given that there are $\binom{w}{s-1}$ ways to sample the $s - 1$ integers, the distance d between the cut and the first sampled point is then distributed as $\frac{\binom{w-d}{s-2}}{\binom{w}{s-1}}$. As this analysis is symmetric for any "cut," we claim that the distribution of all G_i are identical. □

While the analysis above is conditioned on the case where we have s uniformly random chosen positions, the number of sampled positions varies across windows and is only lower-bounded by s. If we replace s with the expected number of minmers in the window, d_f, we can obtain an approximation of the distribution of distances (Figure 2). A more rigorous analysis, which is beyond the scope of this work, would require a distribution for the number of sampled positions in a window rather than just the expectation.

Unfortunately, this distribution is not that useful on its own. Given that the distribution of the distance is the same across all points, we have that $(s + 1)\,\mathbb{E}[G_i] = w + 1$ and therefore $\mathbb{E}[G_i] = (w + 1)/(s + 1)$. Even more interesting than the expectation, though, are the order statistics of G_i, such as $\max G_i$.

In *Order Statistics* David and Nagaraja (2004), a similar problem is studied where a rope of length 1 is cut at n randomly selected locations. The authors show that the expected length of the longest segment is $H_{n+1}/(n + 1)$, where H_n is the nth harmonic number. The details of the problem we describe above are slightly different, as the "cut-points" are selected from a set of integers without replacement as opposed to sampled from $[0, 1]$. We can use this to define $\bar{\mathcal{G}}_i$, an estimator for $\max G_i$,

$$\bar{\mathcal{G}}_i = (w + 1)\frac{H_{d_f+1}}{d_f + 1}$$

As w grows, the effect of sampling without replacement grows smaller and the error of $\bar{\mathcal{G}}_i$ becomes solely from the fact that d_f is only an expectation of the number of minmers in a window.

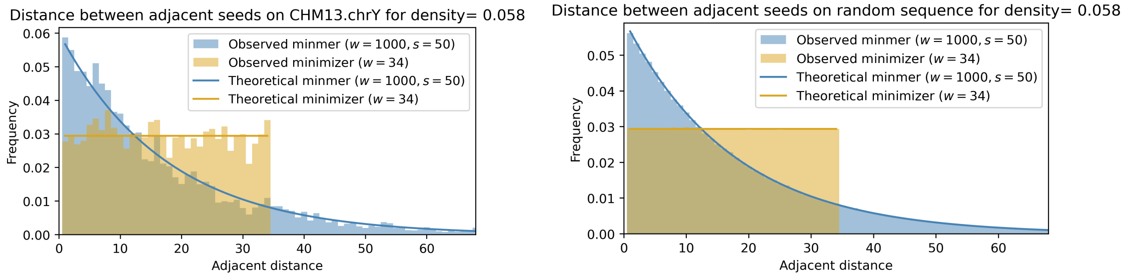

(a) (b)

Supplementary Figure 2: **Simulated and empirical spread.** The spread of minmers and minimizers under similar densities on the human Y-chromosome (a) and a simulated random sequence (b).

1.6 Median prediction error for simulated sequences

If an unbiased Jaccard estimator is generated from a symmetric distribution, then the expected median Jaccard and mean Jaccard would be identical. More importantly, the median ANI would also be unbiased, whereas the mean ANI would not necessarily be unbiased.

While the parameters used to replicate the experiments for Table 1 in Belbasi *et al.* (2022) yield a fairly symmetric hypergeometric distribution for the minhash, when the true Jaccard between two sequences is very close to 0 or 1 or the sketch size is decreased, the asymmetry in the hypergeometric distribution is increased, resulting in notable discordance between the mean and median of the Jaccard estimator. In these cases, an unbiased predictor of the mean Jaccard is not necessarily an unbiased predictor of the median Jaccard and therefore also not an unbiased predictor of the median ANI.

The median results for the replicated Belbasi *et al.* (2022) experiment as well as the median results for the simulated read mappings can be found in Supplementary Figure 3 and Supplementary Table 1, respectively.

	Minimap2	MashMap2	MashMap3
Dataset	Median error	Median error	Median error
CLR-99	**0.00**	-0.25	0.05
CLR-98	**0.02**	-0.31	0.10
CLR-95	**0.06**	0.20	0.27

Supplementary Table 1: **Median error for ANI estimates of simulated human Nanopore reads.** Minmer and minimizer-based MashMap implementations as well as Minimap2 were used to map simulated reads from the human reference genome using Pbsim Ono *et al.* (2013).

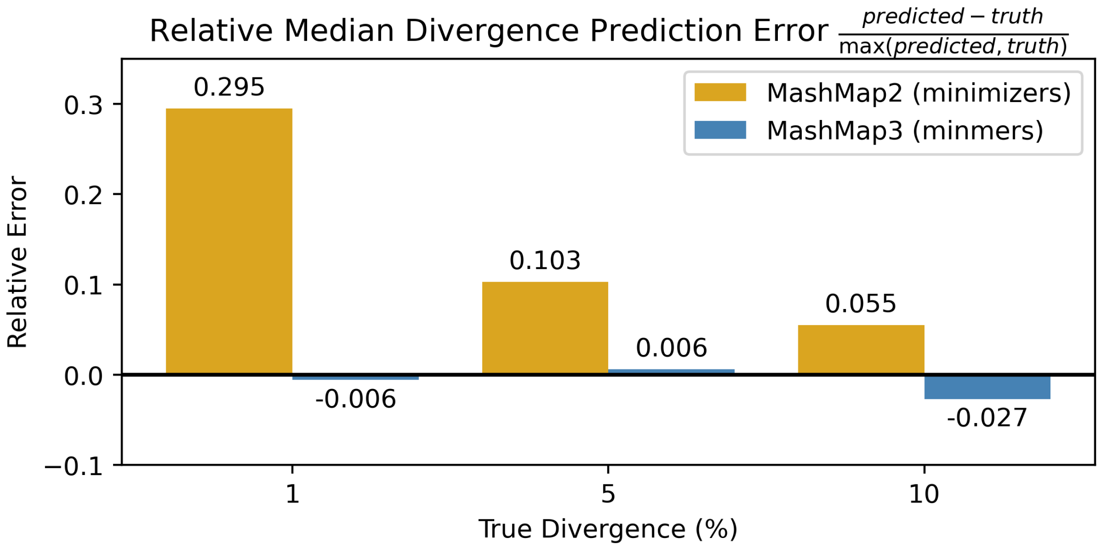

Supplementary Figure 3: **Median error on ideal sequences.** Experiments from Belbasi *et al.* (2022) were replicated with MashMap3, and the relative median prediction error is reported.

1.7 Indexing requirements across different parameters

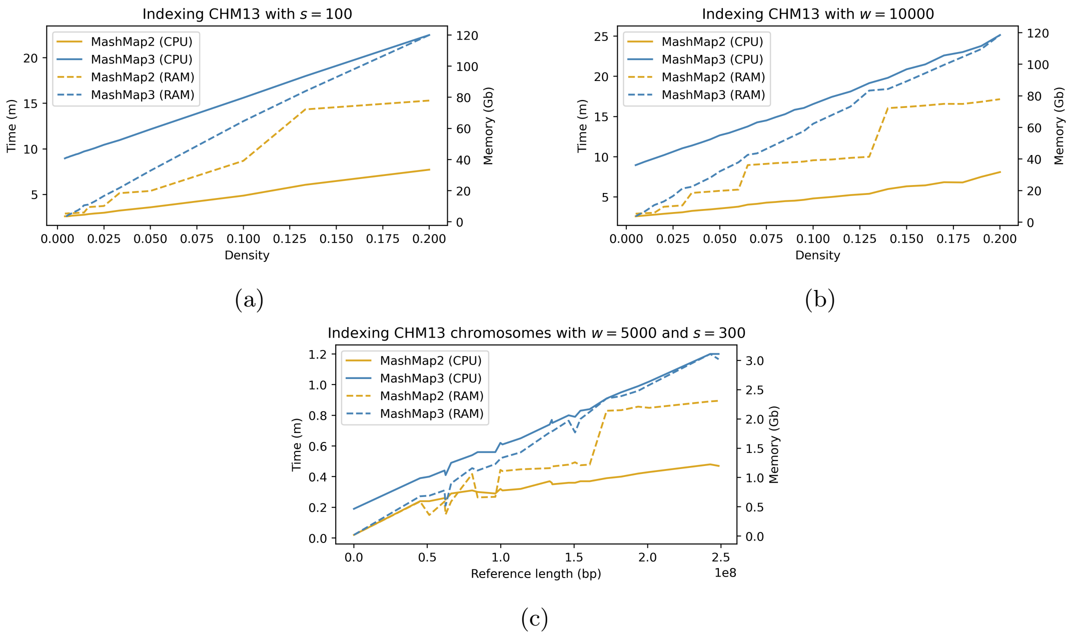

Supplementary Figure 4: **Indexing the CHM13 assembly with different parameters.** In (a), the window length was changed while holding the sketch size at $s = 100$, resulting in a range of densities from 0.01 to 0.20. Similarly, in (b) the window length was held at $w = 10000$ while the sketch size was varied to generate sketches with densities from 0.01 to 0.20. In (c), performance benchmarks were generated from indexing each of the CHM13 chromosomes separately with $w = 5000$ and $s = 300$.

1.8 Simulated read results and the effects of indels

Difference Ratio	CLR-95 ME	CLR-98 ME	CLR-99 ME
20:40:40	0.30	0.11	0.05
100:00:00	0.00	-0.02	-0.02

Supplementary Table 2: **The effect of indels on ANI prediction error.** For error rates of 1%, 2%, and 5%, Pbsim was used to generate two datasets, one with a mismatch, insertion, deletion ratio of 20:40:40 and another with mismatches only (100:00:00). ANI was estimated from the Jaccard using the binomial model.

1.9 ANI prediction performance on low-complexity queries

		MashMap2			MashMap3		
Query species	ANI threshold	Basepairs mapped (Gbp)	Mean error	Mean absolute error	Basepairs mapped (Gbp)	Mean error	Mean absolute error
chimpanzee	95%	0.01	**0.76**	**1.36**	0.01	1.05	1.51
chimpanzee	90%	0.03	4.51	4.76	0.03	**4.43**	**4.63**
chimpanzee	85%	0.04	4.85	5.11	0.04	**4.81**	**5.03**
macaque	95%	<0.01	**0.63**	1.66	<0.01	0.86	**1.55**
macaque	90%	<0.01	2.13	2.96	<0.01	**0.72**	**1.74**
macaque	85%	0.05	9.79	9.88	**0.08**	**7.98**	**8.03**

Supplementary Table 3: **Proportion and accuracy of low-complexity mappings.** MashMap2 and MashMap3 were used to align the human reference genome to chimpanzee and macaque genomes. The number of aligned query query nucleotides from low-complexity segments as well as the mean error and mean absolute error of the mappings are reported here.

References

Belbasi, M. *et al.* (2022). The minimizer jaccard estimator is biased and inconsistent. *Bioinformatics*.

Comtet, L. (1974). *Advanced Combinatorics: The art of finite and infinite expansions*. Springer Science & Business Media.

David, H. A. and Nagaraja, H. N. (2004). *Order statistics*. John Wiley & Sons.

Jain, C. *et al.* (2018). A fast adaptive algorithm for computing whole-genome homology maps. *Bioinformatics*, **34**(17), i748–i756.

joriki (2012). Distribution probability of elements and pair-wise differences in a sorted list. Mathematics Stack Exchange. URL:https://math.stackexchange.com/q/247409 (version: 2012-11-30).

Ono, Y. *et al.* (2013). Pbsim: Pacbio reads simulator—toward accurate genome assembly. *Bioinformatics*, **29**(1), 119–121.
